# Bilateral orchidopexy for intermittent testicular torsion

**DOI:** 10.1002/bco2.439

**Published:** 2024-09-20

**Authors:** Paul K. Hegarty, Mona Kalantar, Penelope A. Hegarty, Helen Zafirakis, Jack E. Monahan

**Affiliations:** ^1^ Consultant Urologic Surgeon Mater Private Network Dublin Ireland; ^2^ Faculty of Medicine University College Cork Cork Ireland; ^3^ Consultant Urologic Surgeon Bon Secours Hospital Cork Ireland

**Keywords:** intermittent, orchidopexy, outcomes, testis, torsion

## Abstract

**Objectives:**

To assess the effect of bilateral orchidopexy in preventing future torsion and testicular loss in patients with intermittent testicular torsion. Secondarily, this study aims to assess the rate of pain improvement following orchidopexy.

**Methods:**

This is a prospective cohort of patients. Participants were men who underwent elective bilateral orchidopexy for intermittent testicular torsion. All consecutive cases were treated by a single surgeon in a single centre between 2015 and 2023. The primary outcomes were prevention of torsion and testicular loss. The secondary outcome was the resolution or improvement in pain.

**Results:**

The success rate of bilateral orchidopexy in preventing testicular loss due to torsion was 100%, at a follow‐up of mean 33.5 months. Of the 50 patients, 88% were pain‐free following orchidopexy, and 12% had an improvement in their pain. There were no cases of hydrocoele or haematoma in this series. To our knowledge, this is the largest series reported in the literature.

**Conclusions:**

Elective bilateral orchidopexy prevents torsion and preserves testicular viability. Pain is resolved in most but not all cases. This is important in counselling men who are considering surgical management of intermittent torsion of the testicle.

## 1 INTRODUCTION

Testicular torsion occurs because of rotation of the testis resulting in the twisting of the spermatic cord and ultimately compromising blood flow to the testis.^1^ It can occur at any age but is most common in young males.^2^ It is estimated that the incidence of testicular torsion is four per 100 000 males under the age of 25 years; however, a 10‐year analysis in Ireland showed a much higher incidence of 22 per 100 000.^3^ Acute testicular torsion is a urological emergency with surgical exploration and orchidopexy considered the mainstay of treatment.^4^ While acute testicular torsion is well studied and described in the literature, there is a paucity of data on intermittent testicular torsion (ITT).^5^

Patients who experience ITT describe episodes of testicular pain that resolve spontaneously.^6^ These episodes are spaced with extended pain‐free periods. As many as 50% of patients with acute testicular torsion have reported experiencing at least one prior episode of testicular pain, believed to be caused by ITT.^5^ ITT puts patients at risk for acute testicular torsion and testicular loss.^7^

The diagnosis of ITT is purely clinical.^1^ The classical history of intermittent torsion is recurrent episodes of scrotal pain spread among intervals of symptom‐free periods. This pain is sometimes associated with a change in the position of the testis.^8^ Physical examination often reveals a change in lie of the testis, resulting in a horizontal lie instead of the usual vertical orientation. The affected testis may also twist along its longitudinal axis, resulting in twisting of the spermatic cord. Examination also reveals hypermobile testes in many cases of torsion, as well as joint hypermobility.^8^, ^9^, ^10^ Ultrasound imaging can often be falsely reassuring as torsion may have resolved.^7^

In cases of ITT, patients are offered conservative and surgical management. Conservative care involves scrotal support with the goal of keeping the testes in a comfortable position and decreasing the incidence of retorsion.^5^ The patient is instructed to attend the nearest emergency department (ED) for urgent attention in the case of severe/unremitting pain. Surgical treatment via bilateral orchidopexy is often recommended in order to reduce the risk of testicular loss in the future. This study was designed to assess the effect of bilateral orchidopexy in the preservation of the testes, as well as improvement in pain.

## 2 PATIENTS AND METHODS

This is a prospective study of 50 men who underwent bilateral orchidopexy for treatment of ITT consecutively between 1 January 2015 and 3 January 2023 by a single surgeon in a single centre. All patients consented to the storage of data on a database. Data was anonymised by the first author. This was analysed by the second author, following institutional review board (IRB) approval from the University College Cork, Ireland. There were no exclusion criteria.

**FIGURE 1:**
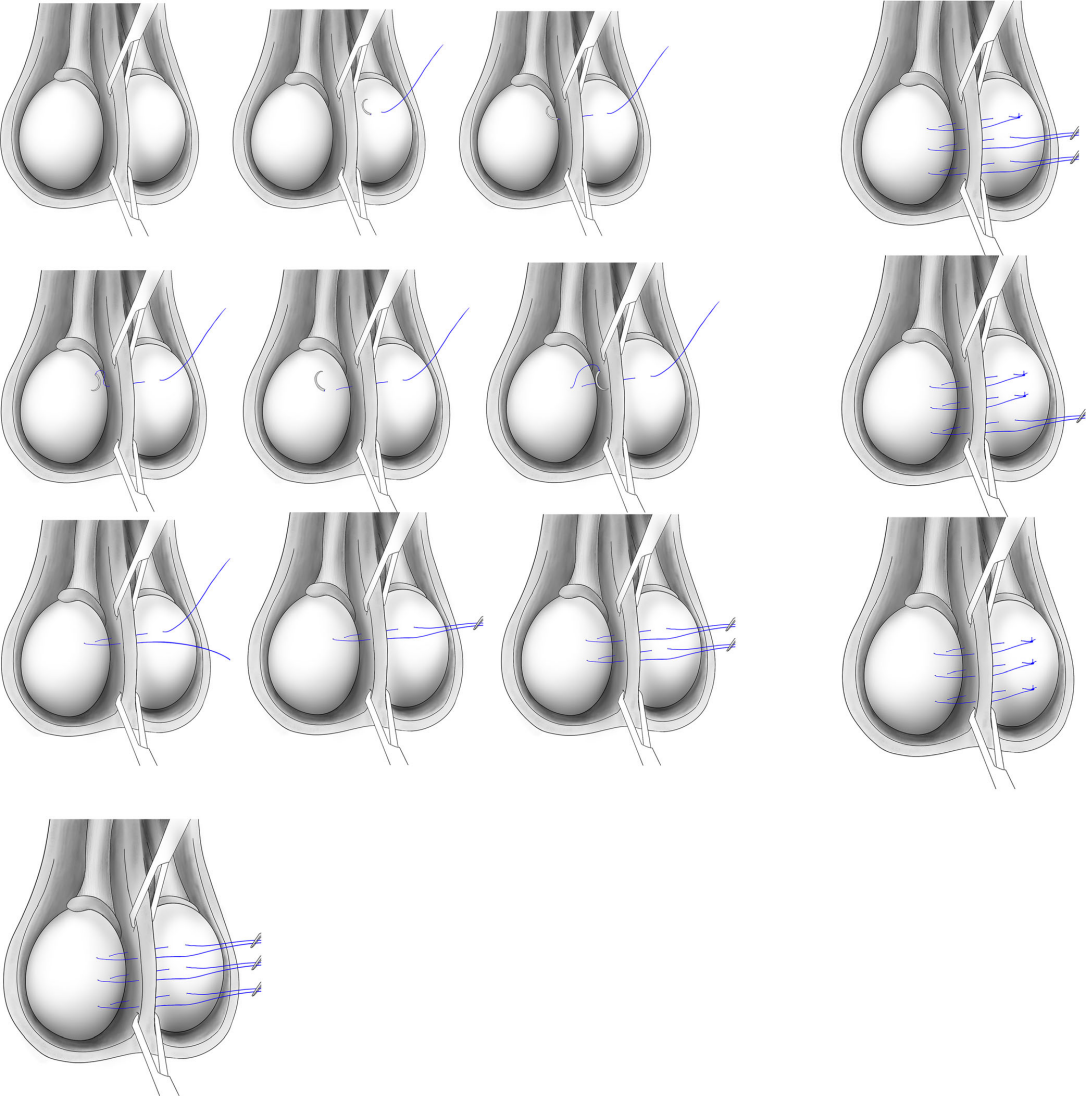
Median mutual fixation of testes using three vertical mattress non‐absorbable sutures.

The surgical technique was the same in all cases. Under general anaesthetic, the patient was placed flat supine. Prophylactic antibiotics were not used. Bilateral spermatic cord blocks were performed using 10 mL levobupivacaine 0.5%. A midline scrotal incision was made through the median raphe. Testes were delivered, structures examined, and any anomalies or abnormal lie noted. The normal position was defined as the epididymis posteriorly with the vas entering towards the lower pole, with no twist of the spermatic cord. Usually, the tunica vaginalis can be preserved intact; however, if there is uncertainty, the tunica vaginalis may be opened. Once content with the orthotopic position of the testes, they are sutured medially to each other through the septum as vertical sutures, using 2/0 polypropylene sutures, three times (Figure 1). Sutures are left with 1–2 cm laxity to allow for inferior migration of the testes in later years. Dartos and skin are closed with absorbable sutures and wound glue applied. The patient is advised to wear supportive underwear or scrotal support for at least a week after surgery. Patients were assessed in the clinic by the same surgeon typically 6 weeks after surgery and thereafter.

The following data were collected from the database: age, duration of symptoms, follow‐up duration, side of pain, presence of abnormal testicular lie, presence of testicular atrophy, nausea, presence of longitudinal twist of the testis, presence of hypermobile testes, presence of hypermobile joints, previous ED attendance due to testicular pain, pain outcome after treatment and post‐operative complications. For each individual, the above variables were collected and coded into a spreadsheet.

Statistical analyses were performed using bivariate analyses, Fisher–Freeman–Halton exact tests and Mann–Whitney U tests. Analysis was performed with a confidence interval set at 95%. Data are presented as means ± standard deviation, where applicable. All analyses were performed using IBM SPSS Statistics for Windows, Version 29 (Armonk NY: IBM Corp).

## 3 RESULTS

Fifty males underwent bilateral orchidopexy for ITT. The mean age at the time of the orchidopexy procedure was 28.9 years old (standard deviation 9.7, ranging from 16 to 49 years). Patient characteristics are summarized in Table 1. There was equal presentation of pain left (36%) and right (36%), with 28% of men reporting bilateral pain. A total of 34% had attended the ED previously and 34% described nausea associated with the pain.

**TABLE 1:** Characteristics of patients with intermittent testicular torsion who underwent bilateral orchidopexy.

	*N* (%)
Total	50 (100)
Side of testicular pain	
Left	18 (36.0)
Right	18 (36.0)
Bilateral	14 (28.0)
Previous ED Attendance	
Yes	17 (34.0)
No	33 (66.0)
Nausea	
Yes	17 (34.0)
No	33 (66.0)

	Mean (SD)
Age (years)	28.86 (9.67)
Duration of symptoms (months)	31.55 (52.46)
Duration of follow‐up (months)	33.54 (25.08)

Abbreviation: ED, emergency department.

No case was lost to follow‐up, which was for a mean of 33.5 months.

**FIGURE 2:**
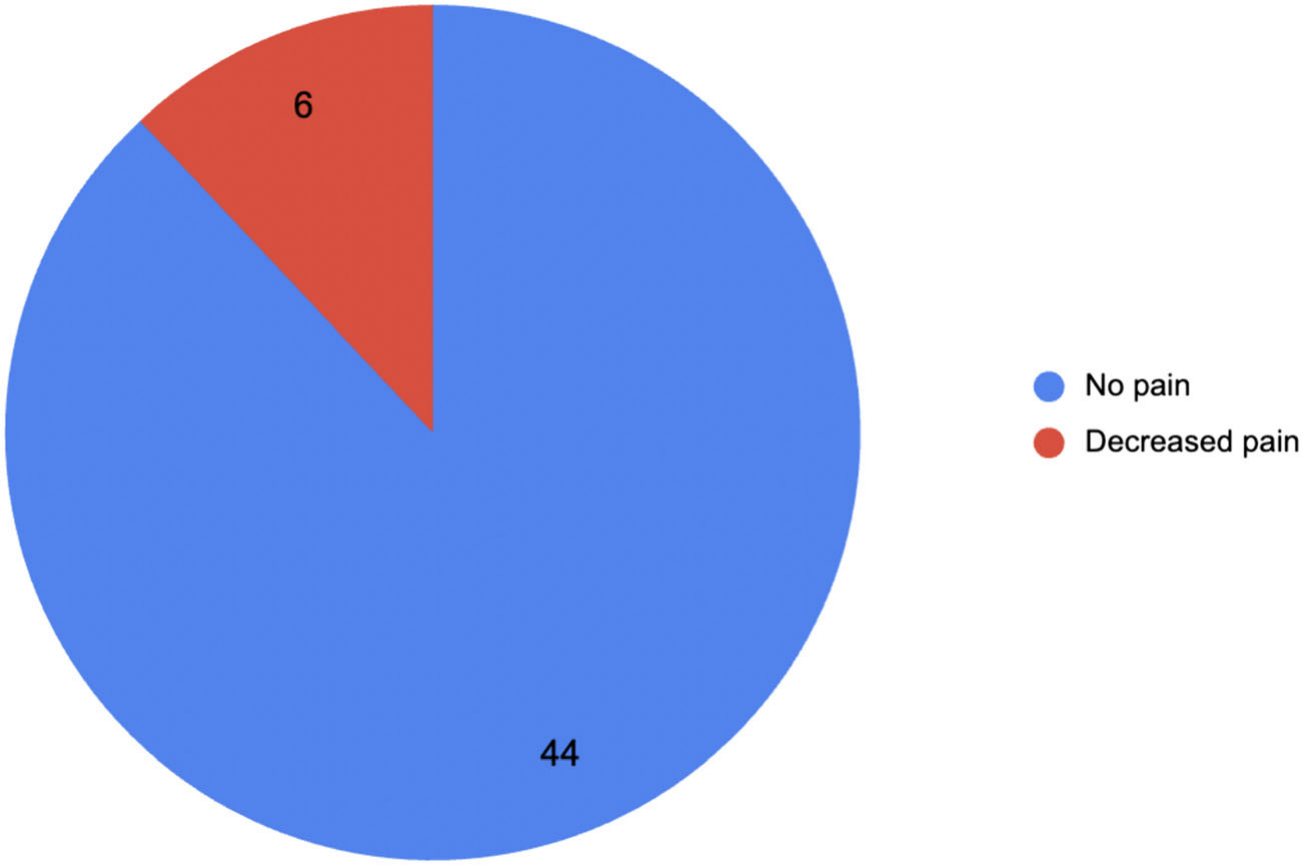
Incidence of pain outcomes following bilateral orchidopexy.

The success rate of bilateral orchidopexy in preventing testicular loss due to torsion was 100%. Figure 2 illustrates the outcome of patients' pain following orchidopexy. A total of 44 men (88%) described full resolution of pain; 6 men (12%) described improved pain. No case described their pain as unchanged or increased. Testicular atrophy was not seen in any patients with ITT prior to surgery nor during the follow‐up period.

Three patients (6%) reported complications. These were superficial wound infections, which resolved fully with a single course of oral antibiotics.

100% of the patients were noted to have testicular hypermobility before their operation.

Joint hypermobility was noted in 18 (36%) of patients. The duration of follow‐up was significantly shorter in patients who were noted to have joint hypermobility versus those who did not (23.00 ± 21.06 and 39.47 ± 25.50, respectively; *p* = 0.033). There was no significant difference in the age between patients who were noted to have joint hypermobility and those who did not (27.56 ± 8.69 and 29.59 ± 10.24, respectively; *p* = 0.498). Similarly, there was no significant difference in the duration of symptoms between patients who were noted to have joint hypermobility and those who did not (34.29 ± 62.03 and 30.00 ± 47.28, respectively; *p* = 0.587).

There was no statistically significant difference in the duration of follow‐up between patients who experienced complications and those who did not (43.00 ± 31.58 and 32.94 ± 24.91, respectively; *p* = 0.567). Similarly, there was no statistically significant difference in age between patients who experienced complications and those who did not (28.67 ± 10.02 and 28.87 ± 9.76, respectively; *p* = 0.902). There was also no statistically significant difference in the duration of symptoms between patients who experienced complications and those who did not (4.67 ± 2.31 and 33.39 ± 53.76, respectively; *p* = 0.073). The presence of complications was not correlated with patients reporting a pain‐free outcome (*p* = 0.509).

The duration of symptoms prior to receiving bilateral orchidopexy was not associated with the age of the patient (*p* = 0.335). There was no statistically significant difference in the duration of symptoms between patients who attended the ED for testicular pain and those who did not (39.00 ± 63.43 and 27.71 ± 46.51, respectively; *p* = 0.820). Furthermore, there was no statistically significant difference in the duration of symptoms between patients with abnormal lie of the testicle and those that did not experience abnormal lie (26.74 ± 31.86 and 39.58 ± 71.96, respectively; *p* = 0.960).

There was no statistically significant difference in the duration of symptoms between patients who experienced longitudinal twist of the testicle versus those who did not (46.33 ± 80.41 and 24.63 ± 31.75, respectively; *p* = 0.493).

The duration of follow‐up after orchidopexy was not correlated with the age of the patient (*p* = 0.559) nor the duration of symptoms (*p* = 0.209). There was no significant difference in the duration of follow‐up between patients with abnormal lie of the testicle and those that did not experience abnormal lie (31.90 ± 26.75 and 25.69 ± 20.68, respectively; *p* = 0.460). Similarly, no significant differences in the duration of follow‐up were seen between the patients with longitudinal twist of the testicle and those that did not experience longitudinal twist (27.67 ± 12.79 and 36.06 ± 26.25, respectively; *p* = 0.434).

## 4 DISCUSSION

This study found that the primary goal of preventing testicular loss in patients with ITT via bilateral orchidopexy was achieved in 100% of cases. This is comparable to a 100% testicular preservation rate following bilateral orchidopexy for ITT in a smaller series of paediatric cases.^6^ While failure of bilateral orchidopexy for testicular torsion is uncommon, retorsion following fixation of the testicle has been documented, in particular when absorbable suture is used.^11^

The surgical technique of suturing testes to each other through the septum is at odds with the technique recommended in the BURST‐BAUS consensus document for the management of acute testicular pain.^12^ The technique of mutual fixation seems better suited to the elective scenario of ITT. The relative lower morbidity of such a technique is reflected in the absence of complications such as hydrocele and haematoma.

Of the 50 patients, no patients reported worsening of their pain following orchidopexy. An improvement or complete resolution of symptoms was found in all patients who underwent surgery. Six (12%) patients reported an improvement in their pain while 44 (88%) patients reported complete resolution of pain. These figures are in line with the data presented in existing research, which suggests resolution or improvement of pain in 88%–100% of patients.^5^, ^6^, ^7^, ^13^ This is important in managing the expectations of patients considering surgery. The main aim is to protect testicular viability, whereas pain may not resolve fully in all cases. Those with persistent pain may have other causes of pain, or the technique is insufficient to relieve pain in all cases. This is important in order to set realistic expectations for patients considering surgical management.

Three patients (6%) in this study experienced superficial wound infection following orchidopexy. Series of orchidopexy for cryptorchidism report complication rates are 0%–10%.^4^, ^7^, ^14^, ^15^ Although being of a similar rate, the complications in this study are significant and are a focus for change in practice. This should be addressed such as by reassessment of the quality of surgical hand antisepsis, wound dressings, and antimicrobial prophylaxis.^16^, ^17^, ^18^, ^19^ Antibiotic stewardship aims to limit the development of resistant organisms for individuals and the community. It is debatable as to whether this rate of superficial skin infection justifies giving antibiotic prophylaxis for the group as a whole.

The side of testicular pain due to torsion observed in the cohort of patients involved in this study was 18 (36%) with left‐sided testicular pain, 18 (36%) with right‐sided pain, and 14 (28%) of patients with bilateral testicular pain. This differs from other series where the left testicle is predominant.^2^, ^8^, ^20^

Testicular atrophy was not seen in any of the patients prior to orchidopexy in this cohort. Sellu and Lynn^21^ found that 41.7% of their patients with ITT experienced testicular atrophy, while Kamaledeen and Surana^22^ identified atrophy in 25% of patients with ITT. This inconsistency in the rates of testicular atrophy prior to orchidopexy may be due to several factors. Firstly, assessing testicular atrophy via clinical examination is difficult, particularly in the paediatric population where the boy is still undergoing growth. Furthermore, the studies had only 8 and 12 subjects, respectively. Our study was limited in that atrophy was assessed clinically by the single surgeon in consultation with the patient, which is open to bias.

A study investigating testicular atrophy following acute testicular torsion found that 41.4% of patients had some degree of atrophy on ultrasound in follow‐up.^2^ This difference may be due to greater sensitivity of ultrasound or may reflect different populations between acute testicular torsion and ITT.

Strengths of this paper include the sample size of this study and the duration of the study. The sample size is significantly larger sample size when compared with all known studies in the adult population. As ITT is an underreported condition, it makes obtaining a large sample size challenging. A multicentre study may improve recruiting numbers and reduce the bias of a single‐site study. The mean follow‐up time was 33.54 in this study. This is longer than other papers, which have a follow‐up of 4–8 months.^5^, ^7^ No cases were lost to follow‐up. This suggests long‐term durable outcomes in terms of testicular preservation and resolution of pain.

Limitations of this study include being from a single surgeon in a single centre over an 8‐year period. Furthermore, ITT is a clinical diagnosis with no diagnostic validated test. This makes it less certain that all the patients diagnosed were true ITT and may account for the persistent pain in some cases.

## 5 CONCLUSION

This study found the success rate of bilateral orchidopexy to be 100% in preventing future torsion and testicular loss. Improvement or complete resolution of symptoms was also found in 100% of patients. Bilateral testicular fixation appears to be effective in the management of intermittent torsion of the testicle.

## AUTHOR CONTRIBUTIONS

*Design*: Paul K. Hegarty and Helen Zafirakis. *Data collection*: Mona Kalantar and Paul K. Hegarty. *Writing*: Paul K. Hegarty, Mona Kalantar and Jack E. Monahan. Illustrations: Penelope A. Hegarty and Helen Zafirakis. *Proofreading*: Paul K. Hegarty.

## CONFLICT OF INTEREST STATEMENT

None of the authors have a conflict of interest in this article.
